# Zerumbone from *Zingiber zerumbet* (L.) smith: a potential prophylactic and therapeutic agent against the cariogenic bacterium *Streptococcus mutans*

**DOI:** 10.1186/s12906-018-2360-0

**Published:** 2018-11-13

**Authors:** Thiago Moreira da Silva, Carlos Danniel Pinheiro, Patricia Puccinelli Orlandi, Carlos Cleomir Pinheiro, Gemilson Soares Pontes

**Affiliations:** 1 Instituto Nacional de Pesquisa da Amazônia, Coordenação de Tecnologia e Inovação, Av. André Araújo – 2936 – Petrópolis, Manaus, Amazonas 69067-375 Brazil; 2 Instituto Leônidas e Maria Deane, Fundação Oswaldo Cruz, Rua Teresina, 476 - Adrianópolis, Manaus, AM 69057-070 Brazil; 3 Instituto Nacional de Pesquisa da Amazônia, Coordenação Sociedade, Ambiente e Saúde, Av. André Araújo – 2936 – Petrópolis, Manaus, 69067-375 Amazonas Brazil

**Keywords:** Tooth decay, Zerumbone, Bioprospecting, Antimicrobial, Treatment, Phytotherapy

## Abstract

**Background:**

Essential oil obtained from rhizomes of the *Zingiber zerumbet* (L.) Smith (popularly known in Brazil as bitter ginger) is mainly constituted by the biomolecule zerumbone, which exhibit untapped antimicrobial potential**.** The aim of this study was to investigate the antimicrobial activity of the zerumbone from bitter ginger rhizomes against the cariogenic agent *Streptococcus mutans*.

**Methods:**

Firstly, the essential oil from rhizomes of *Zingiber zerumbet* (L.) Smith extracted by hydrodistillation was submitted to purification and recrystallization process to obtain the zerumbone compound. The purity of zerumbone was determined through high-performance liquid chromatography analysis. Different concentrations of zerumbone were tested against the standard strain *S. mutans* (ATCC 35668) by using microdilution method. The speed of cidal activity was determined through a time kill-curve assay. The biological cytotoxicity activity of zerumbone was assessed using Vero cell line through MTT assay.

**Results:**

The zerumbone showed a minimum inhibitory concentration (MIC) of 250 μg/mL and a minimum bactericidal concentration (MBC) of 500 μg/mL against *S. mutans.* After six hours of bacteria-zerumbone interaction, all concentrations tested starts to kill the bacteria and all bacteria were killed between 48 and 72 h period at the concentration of 500 μg/mL (99,99% of bacteria were killed in comparison with original inoculum). In addition, zerumbone showed no cytotoxicity activity on mammalian continuous cells line.

**Conclusions:**

These results draw attention to the potential of zerumbone as antimicrobial agent against *S. mutans* infection, indicating its possible use in the phyto-pharmaceutical formulations as new approach to prevent and treat tooth decay disease.

**Electronic supplementary material:**

The online version of this article (10.1186/s12906-018-2360-0) contains supplementary material, which is available to authorized users.

## Background

Several medicinal plants contain in their biochemical constitution many compounds with antibacterial action, that remains unknown [1]. *Zingiber zerumbet* (L.) Smith, a rhizomatous herbaceous species, belonging to the family *Zingiberaceae,* is a native plant from Southeast Asia with potential antimicrobial activity not fully comprehended [2]. In Brazil, this species is usually identified as bitter ginger and is easily found in large numbers in the Amazonas state, where it is well adapted to the local climatic conditions.

**Fig. 1:**
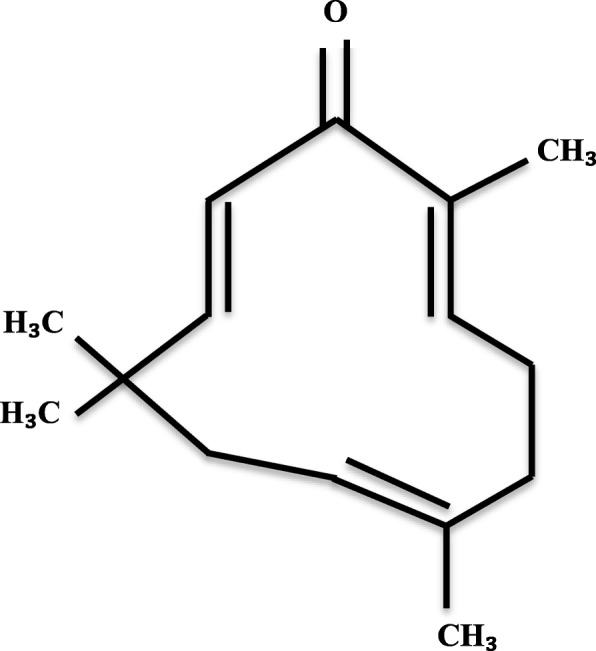
Chemical structure of zerumbone

Essential oils extracted from *Zingiber zerumbet* rhizomes have potential pharmacological activities, including antimicrobial, anti-inflammatory, chemo-preventive, antinociceptive, antiulcer, antioxidant, antipyretic and analgesic, as previously described [3–7]. The major bioactive molecule found in the essential oil of *Zingiber zerumbet* rhizomes is the zerumbone (Fig. 1), a monocyclic sesquiterpene compound (2,6,10-cy-cloundecatrien-1-one, 2,6,9,9-tetramethyl-,(E,E,E)-) [8]. Zerumbone has been linked to a broad range of biological activities, including the antibacterial action [9, 10].

Previous reports have demonstrated the antimicrobial activities of zerumbone against Gram negative bacteria, such as *Escherichia coli* and *Helicobacter pylori*, and Gram positive bacteria, such as *Staphylococcus epidermidis* and *Staphylococcus aureus*, showing more effectiveness on Gram positive microorganisms [7, 11]. However, its activity against other Gram positive microorganisms such as *Streptococcus mutans* is unknown. *S. mutans* is the main causative agent of tooth decay, the oral infectious disease most prevalent in the world affecting over 90% of school-aged children and about 100% of the world population [12].

Despite the great diversity of bacterial species in the oral cavity, few are able to cause tooth decay (cariogenic bacteria) and *S. mutans* has been implicated as the major etiological agent of this oral infectious disease [13]. The cariogenic potential displayed by this bacterium is due to its ability to produce acid (acidogenic) from dietary carbohydrate, capacity to survive in low-pH environments (aciduric) and, especially, due to its great ability to adhere onto the tooth surfaces, which makes *S. mutans* responsible for the initial formation of dental plaque [14].

Chemicals agents such as chlorexidin and others phenolic compounds are available and can be used to prevent tooth decay, but long term use of these compounds may result in side effects like loss of taste, metallic taste in mouth, dental pigmentation, diarrhea and oral burning sensation [15]. In this context, biomolecules isolated from plants have been suggested as alternative therapeutics over synthetic chemical agents for prevention of tooth decay, because of their few or no side effects [16]. Hence, the main goal of this study was to investigate the antimicrobial activity of the zerumbone obtained from rhizomes of *Zingiber zerumbet* (L.) Smith against *S. mutans*, the main etiological agent of tooth decay.

## Methods

### Acquisition of *Zingiber zerumbet* rhizomes

The rizhomes of the *Z. zerumbet* were collected in a rural area surrounding the city of Manaus/AM, located at BR-174, points P01 to P02, latitude 24132, 03789 S and longitude 600931,40854 W, according to geographic coordination. After that, an exsiccate was sent to the herbarium of the National Institute of Amazonian Research (INPA) for proper identification and comparison with the exsiccate previously identified by Prof. Dr. Paul Maas (Department of Plant Ecology and Evolutionary Biology; Herbarium University of Utrecht), which is deposited in the herbarium under N°. 186913.

### Essential oil extraction

The extraction of the essential oil (EO) was carried out in the Thematic Laboratory of Chemistry and Natural Products at INPA. The EO was obtained through the hydrodistillation of rhizomes. Briefly, after the identification, cleaning and disinfection, the material was crushed and dried at room temperature. Clevenger apparatus adapted with a round-bottom flask of 2l volume was used for distillation of OE from the crushed material diluted in distilled water in a proportion 1:4. The extraction was done during 6 h starting at boiling point. Afterward, the EO was collected from the condenser and stored in amber flasks at room temperature. All system was protected from light using aluminum foil. The OE yield was estimated by calculating the ratio between the oil mass and the feed mass.

### Gas chromatography–mass spectrometry (GC–MS) analysis

The GC-MS analysis was performed as previously described, with few modifications [17]. Briefly, the investigation of essential oil composition was carried out using Hewlett Packard HP/série 6890 GC SITEM PLUS gas chromatographer (6511 Bunker Lake Blvd. Ramsey, Minnesota, 55303 USA) with an analytical HP-5MS 5% phenylmethylsiloxane capillary column (30 m × 0.32 mm i.d, film thickness 0,25 μm). It was operated an electron ionization system with ionization energy of 70 V. The analyses were conducted using helium and nitrogen gases at 2 mL/min with purity percentage of 99.999%. It was injected 1 μL in splitless mode, in 1:20 ratio of hexannic solution. The oven was programmed at controlled temperature between 60 and 240 °C, raising 3 °C/min and kept at 250 °C for 10 min. Temperature of mass transfer and injection were established at 220 °C and 290 °C, respectively. The chemical constituents of essential oil analyzed were expressed as relative percentage by peak area normalization. The identification of the OE constituents was done by calculating the retention time obtained in the analyzes of GC-MS, correlating them with the retention times of the n-alkanes (C9-C30). The indices were compared with the data available in the NIST / WILEY library [18]. The zerumbone identification was done as previously described [19].

### High-performance liquid chromatography (HPLC)

After purification and recrystallization of the EO using our patented method (n^0^ PI-0505343-9/28/11/2007), the zerumbone purity was estimated through HPLC analysis (Accela High Speed LC, Thermo Scientific®), using a column Hypersil Gold (50 × 2,1 mm) and a mobile phase methanol:water (85:15, *v*/v) at 1 mL/min. The identification was done by comparing the retention time of the peaks with those standard solutes in HPLC and confirmed by UV-absorption spectrum (~ 252 nm).

### Inoculum standardization

The antimicrobial activity of the zerumbone was evaluated against the standard strain of *S. mutans* ATCC 35668 (American Type Culture Collection, Microbiologics Inc., St. Cloud, USA) [20]. Firstly, bacterial suspensions were prepared by inoculation of colonies into a tube containing 3 mL Brain Heart Infusion (BHI) broth, followed by incubation at orbital shaking of 150 RPM for 72 h at 37 °C in anaerobic conditions, as previously described [21] . After incubation, the turbidity was calibrated and adjusted through spectrophotometer analysis to match the 0,5 MacFarland scale (1 × 10^8^ CFU/mL). Final inoculum of 1 × 10^6^ CFU/ mL was used in the assays.

### Determination of the antimicrobial activity

The antimicrobial activity of zerumbone was evaluated by estimation of Minimum Inhibitory Concentration (MIC) and Minimum Bactericidal Concentration (MBC) using microdilution method according to the guidelines of the Clinical & Laboratory Standards Institute (CLSI) [22]. The working stock solution of zerumbone was prepared in polyethylene glycol sorbitan monolaurate (Tween 20) under vigorous magnetic stirring system to assure homogeneity, as previously described [7]**.** After diluting serially this working stock solution in 96 well-plate containing 100 μL BHI broth, 100 μL of BHI broth having bacterial inoculum (1 × 10^6^ CFU/mL) were added into wells resulting in a final volume of 200 μL, followed by incubation at the same conditions mentioned above**.** The concentrations of zerumbone tested ranged from 125 to 2000 μg/mL. Additional wells containing diluting agent of zerumbone (tween 20 10%) was used as a control.

After incubation, the bacterial growth was evaluated by measuring the turbidity in each well through spectrophotometric analysis (600 nm). Subsequently, an aliquot of 50 μL of each well was collected and seeded on plates containing BHI agar and incubated for 72 h at 37 °C in anaerobic conditions. Next, the plates were analyzed for the presence/absence of bacteria in order to estimate the MIC and MBC. The MIC was estimated according to lowest concentration of zerumbone that inhibits the bacterial growth. The MBC was assessed based on the concentration that kills all viable bacterial cells and therefore reveals no visible bacterial growth on the plates. The number of surviving cells (CFU/mL) was determined through the direct-plate counting technique performed individually by two skilled technicians. The limit of detection used in all tests was 10 CFU. An estimated count was provided in case of countable colonies were presented below this limit, as previous reported [23]. All tests were done in triplicate and repeated three times to verify the reproducibility of results.

### Time-kill curve assay

To determine the speed of cidal activity of the zerumbone, a time kill-curve was performed as previously described, with few modifications [24] . An inoculum of 1 × 10^6^ CFU/mL was added into tubes having 3 mL of BHI broth or BHI broth treated with zerumbone (MIC or MBC values) or tween 20 10% (control) and then incubated for 72 h anaerobically with orbital rotation of 150 RPM at 37 °C. The tube containing only bacteria and BHI broth was used to estimate the different phases of growth curve.

An aliquot of 100 μL was removed at 0, 6, 12, 24, 48 and 72 h time intervals to determine the bacterial growth by measuring turbidity through spectrophotometric analysis (600 nm). Subsequently, the aliquots were serially diluted in 0.85% of sterile saline solution, seeded in BHI agar and then incubated at the conditions set out above. The viable number of bacterial cells was estimated by counting CFU and multiplying the results by dilution factors. Means of duplicate colony counts were taken. To build the time-kill curve, the log_10_ CFU/mL versus time over 72 h was plotted. The decrease of 99.9% (≥ 3 log_10_) of the total number of CFU/mL in the original inoculum was used to estimate the bactericidal activity. The assays were performed in triplicate and repeated three times to confirm the reproducibility of results.

### Cytotoxicity assay

The cytotoxicity activity of zerumbone was determined using the MTT (3-(4, 5-dimethyl thiazol-2-yl)-2, 5-diphenyl tetrazolium bromide) assay as previously described with few modifications [21]. Summarily, Vero cells line (2 × 10^4^ per well) were cultured into 96-well plate containing 0.2 mL of DMEM medium (with 10% FBS, penicillin-streptomycin and amphotericin B) per well, in atmosphere of 5% CO_2_ at 37 °C for 24 h. After formation of sub-confluent monolayer, the cells were treated with zerumbone (25 μg/mL; 50 μg/mL and 100 μg/mL) and incubated again at the same conditions above mentioned for 24 and 48 h. For this assay, crystals of zerumbone were diluted in 100% dimethyl sulfoxide (DMSO) because of its low aqueous solubility. Thus, the maximum concentration of zerumbone containing safe percentage of DMSO (< 1%) to the cells was 100 μg/mL. Sterile PBS was used as positive control and DMSO 100% as negative control. Subsequently, the medium was removed from all wells and 10 μL of MTT (5 mg/mL in sterile PBS) diluted in 100 μL of DMEM medium (without phenol red to avoid misinterpretation) was added into the wells and incubated in atmosphere of 5% CO_2_ at 37 °C for 4 h. After that, the MTT was removed and 50 μL of MTT lysis buffer were added to each well followed for gently homogenization to dissolve the formazan crystals and incubated again for 10 min at the same conditions mentioned earlier. Optical densities of samples were measured using a microplate reader at wavelength of 570 nm. The relative viability of cells was estimated using the following equation: (A570 of treated sample)/(A570 of untreated sample) × 100. All tests were done in triplicate.

### Statistical analyses

Descriptive statistics was used to summarized and describe de data. The results are expressed in mean ± SD.

## Results

### Acquisition of Essential oil and purification of zerumbone

**Fig. 2:**
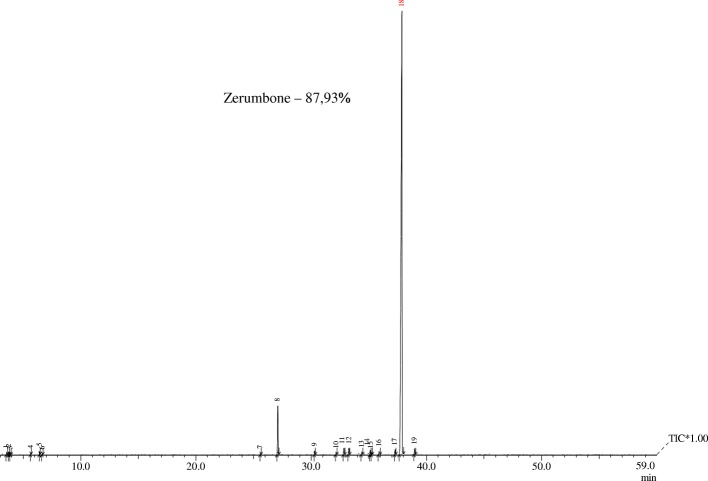
Gas Chromatography-Mass Spectrometry (GC-MS) of Essential oil *Zingiber zerumbet* (L.) Smith. Analysis revealed the presence of 19 compounds. Zerumbone was the major compound (87,93%) found in the essential oil used in this study.* R. time: retention time

**Fig. 3:**
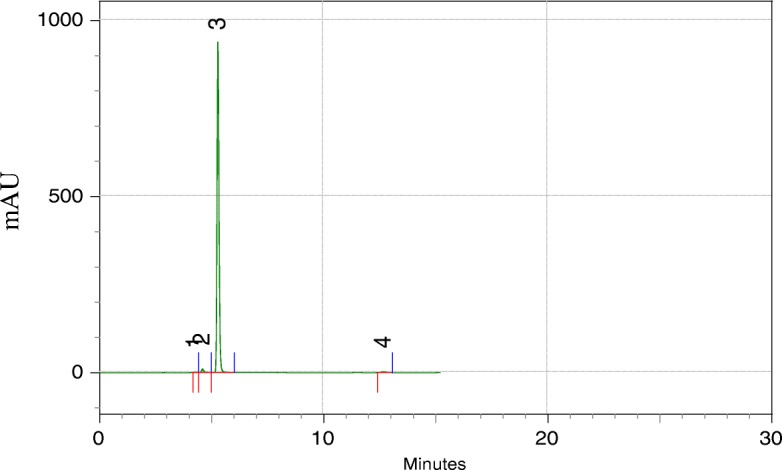
High-performance liquid Chromatography (HPLC) of zerumbone crystals. HLPC elution of zerumbone crystal with retention time of ~ 5 min confirmed zerumbone purity of 98% (peak 3)

The yield of the essential oil obtained was 5%. The EO showed to be constituted mainly by sesquiterpene zerumbone compound, according to GC-MS analysis (Fig. 2). A percentage of 87,93% of zerumbone was detected among the nineteen others chemical constituents. The purification and recrystallization processes applied to EO resulted in a zerumbone crystal with 98% of purity (Fig. 3). These crystals were used in all subsequent tests.

### Antimicrobial activity of zerumbone

**Fig. 4:**
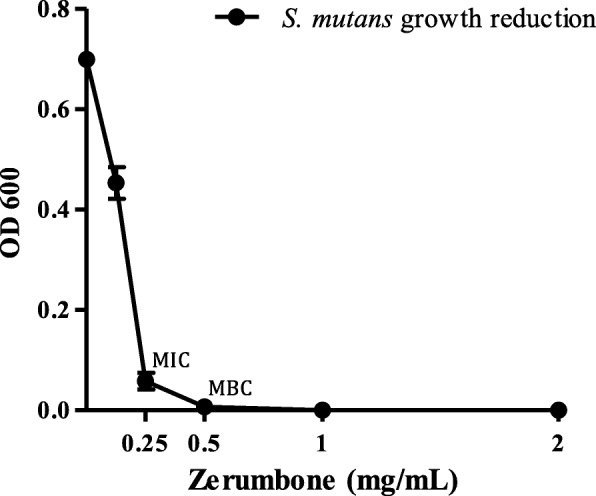
Antimicrobial activity (MIC and MBC) of zerumbone against *S. mutans.* Bacterial inoculum (1 × 10^6^ CFU/mL) was treated with different concentrations of zerumbone and incubated at 37 °C for 48 h in anaerobic conditions. After incubation, the bacterial growth was verified by turbidity measurements using spectrophotometer. The results are representative of three independent experiments performed in triplicate and the values are shown in mean ± SD. OD: optical density; MIC: Minimum Inhibitory Concentration; MBC: Minimum bactericidal concentration

**Fig. 5:**
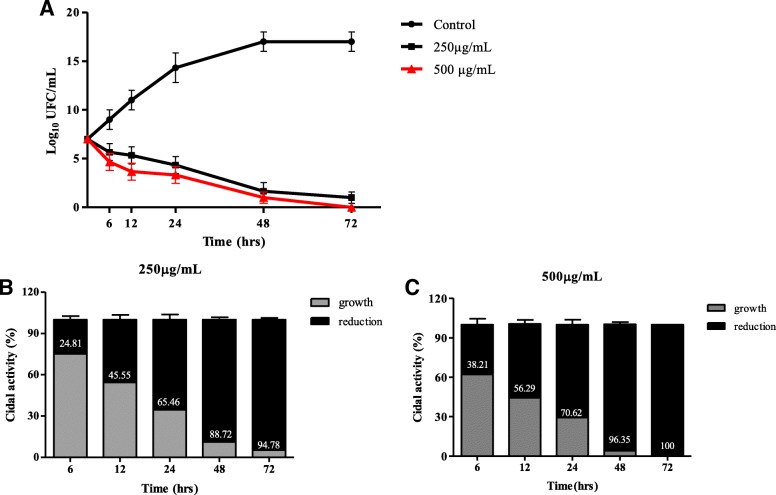
Bacterial- time kill curve. Zerumbone was tested for antimicrobial activity on bacterial-kill kinetics. *S. mutans* were grown in BHI broth + tween 20 10% (control) or along with graded concentrations of zerumbone at 37 °C for 72 h in anaerobic conditions. Samples were collected at different time intervals to estimate bacterial kill kinetics (**a**) and growth reduction (**b** and **c**), by spectrophotometry analysis and CFU counts. Numbers inside of bars at (**b**) and (**c**) figures means percent reduction. Results are representative of three independent experiments and data are expressed in mean ± SD. The interquartile range of each data is indicated by error bars

The zerumbone substance tested in this study demonstrated antimicrobial activity against *S. mutans* ATCC 35668, showing a MIC value of 250 μg/mL and MBC of 500 μg/mL (Fig. 4, Additional file 1: Figure S1). The time-kill curve assay corroborates the antimicrobial activity of zerumbone. This biomolecule displayed more intense antibacterial activity in the time interval 12–48 h, corresponding to log phase of *S. mutans* growth curve (Additional file 2: Figure S2). Within 12 h of bacterial exposure to 250 μg/mL and 500 μg/mL of zerumbone concentrations, viable bacterial cells were reduced in 45.55% and 56,29%, respectively, in comparison to the original inoculum (Fig. 4a). After 24 h of exposure, the reduction was more pronounced with 65.46% (250 μg/mL) and 70.62% (500 μg/mL) of bacterial cells death (Fig. 4b and c). Finally, the zerumbone showed its maximum action in the interval of 48–72 h, reducing 94.78% of bacterial colonies at concentration of 250 μg/mL and killing all bacteria at the concentration of 500 μg/mL (Fig. 5a, b and c).

### Cytotoxicity activity of zerumbone

**Fig. 6:**
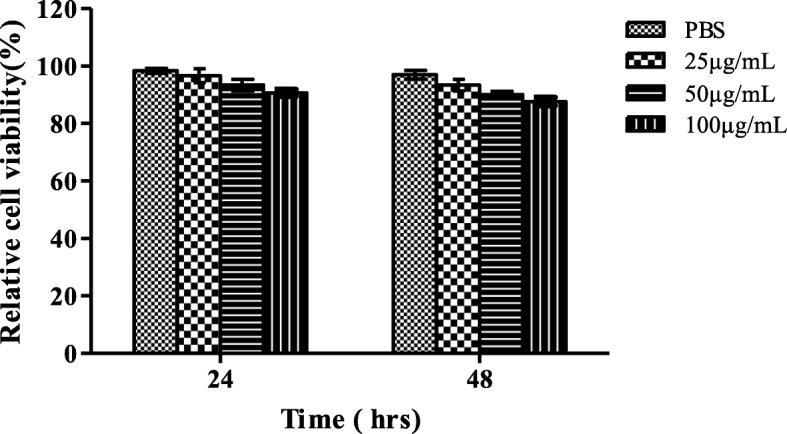
Cytotoxicity of zerumbone against Vero cell line using MTT assay. The data are representative of three independent experiments and the results are expressed in mean ± SD

The MMT assay demonstrated that the biomolecule zerumbone had no considerable cytotoxicity effect up to 100 μg/mL. At concentrations 25, 50 and 100 μg/mL the percentages of cell viability were 100, 97 and 92%, respectively, after 24 h treatment (Fig. 6). The cell viability slightly changed at concentration 50 μg/mL (93%) and 100 μg/mL (87%) after 48 h treatment. The results clearly show that the zerumbone has no cytotoxic effect on normal mammalian cells at the concentrations tested.

## Discussion

Tooth decay disease is a growing global concern that is known to threaten human health and safety [25]. Emerging antibiotic resistance and inconvenient side effects caused by commercial antibiotics usually result in leaving treatment [26]. In this regard, the development of alternative antibiotic therapies based on bioactive molecules from medicinal plants is urgent and crucial to provide effective prevention and treatment for tooth infection.

The present study demonstrates strong antimicrobial activity of zerumbone against the cariogenic agent *S. mutans* (MIC = 250 μg/mL). according to the following classification of antimicrobial action: strong = 50 < MIC< 500 μg/mL moderate = MIC 600 < MIC< 1500 μg/mL and; weak = MIC> 1500 μg/mL [27, 28]. Our data therefore brings to light a new possibility of prophylactic and therapeutic strategies for *S. mutans* infection, especially in case of tooth decay, a serious public health issue [29].

Few reports have been found related to antimicrobial activity of natural biomolecules against the *S. mutans* earlier, but majority demonstrated weak antibacterial action based on the classification set out above [30–32]. A recent study tested around two thousand plant extracts from Amazon region, with seventeen extracts displaying antimicrobial activity against *S. mutans* (ATCC 25175). However, of these, only one extract obtained from plant *Ipomoea alba* L. sp. (*Convolvulaceae*) had strong (MBC ≥ 160 μg/mL) antimicrobial activity [33]. Takarada et al. [34] and Aguiar et al. [35] evaluated the anti-*S. mutans* activity of essential oils from the following plants: *Romarinus officinalis* L., *Melaleuca alternifólia*, *Lavandula officinalis*, *Leptosperfum scoparium*, *Eucalyptus radiate*, *Ageratum conyzoides*, *Artemisia camphorata* Vill., *Bidens sulphurea*, *Foeniculum vulgare* Mill., *Lippia alba*, *Ocimum gratissimum* L., *Pelargonium graveolens*, *Syzygium aromaticum* and *Tagetes erecta* L. The results showed MICs ranging from 500 to 10,000 μg/mL, antimicrobial activities considered weak as compared to our data. Therefore, these studies corroborate the strong bioactive potential of the zerumbone as anticariogenic agent, which could be a good substrate to be used in prophylactic and therapeutic formulations against tooth decay.

Indeed, zerumbone has been tested against a wide range of microorganisms and have showed good antibacterial activity, mainly against gram positive bacteria [36]. But, until now, there is no report describing the antimicrobial activity of the zerumbone or its analogs against *S. mutans*. Hasan et al. [37] demonstrated that extracts from *Zingiber officinale*, species that belongs to same genus and family of bitter ginger, has anti-*S. mutans* action with MIC value of 256 μg/mL, effect similar to the one observed in this study. However, unlike our study, no compound was isolated or purified to determine which biomolecules were responsible for the observed antimicrobial activity. Generally, the essential oil obtained from *Zingiber zerumbet* shows in its constitution a range of zerumbone concentration starting from 12 to 73% [8]. Nevertheless, the essential oil obtained in our study showed 87,93% of zerumbone, which was raised to 98% after purification and recrystallization process using a method patented by our group (n^0^ PI-0505343-9/28/11/2007). This degree of purity is greater than the ones described elsewhere, which allow us to better characterize the zerumbone antimicrobial action [9].

Since zerumbone exhibited efficient antimicrobial activity, we next evaluated the speed of cidal activity. The effectiveness observed was concentration and time-dependent, although both concentrations tested showed similar results. The intensity of zerumbone antimicrobial activity was pronounced during the logarithmic growth phase. After 24 h of bacterial exposure to 250 and 500 μg/mL zerumbone concentrations, viable bacterial cells were reduced to 65.46 and 70.62%, respectively. According to Jones et al. [38] when an antimicrobial agent reduces the bacterial colonies around 70% within 24 h time period, it can be considered as a strong candidate for treating bacterial infections. However, the maximum cidal activity of zerumbone was reached at stationary phase, suggesting the possibility of two mechanism of zerumbone activity, different from the most antibiotic agents that require cell division or active metabolism for the drug’s killing activity [39, 40]. Thus, an antibiotic agent that still shows bactericidal activity under growth-limited conditions may be very advantageous, since the biofilm-associated microorganisms, such as *S. mutans*, may modulate gene expression to enhance endurance under periods of nutrient limitation to survive in the stationary phase [41]. However, further studies are needed to elucidate the antibacterial mechanism of action of zerumbone.

Beyond the determination of the optimum antibacterial concentration, it is also important to guarantee the cytotoxicity safety of the bioactive substances against normal mammalian cells, if the ultimate goal is to use it as a main raw material to produce new drugs. Usually, finding the balance between effectiveness and safety of a biomolecule is very hard mission [42, 43]. Our findings indicated that the zerumbone may not be cytotoxic to normal mammalian cells, since the exposure of Vero cells to different concentrations of zerumbone cause no considerable toxic effects at the concentrations tested. Even after 48 h of zerumbone treatment, no substantial toxic effect was observed, other than a low cytotoxicity evidenced at 100 μg/mL concentration (reduction of 13% of cell proliferation). However, it is imperative to consider that the susceptibility of mammalian cells tend to be greater in the MTT test than in vivo situation, because of the direct exposure of the cells to the biomolecules without any type of variants happening in vivo, such as route of administration and topical absorption, which may influence or even though decrease the cytotoxicity effect demonstrated in vitro [44]. On the other hand, it is important to mention that additional experiments are needed to ensure safe use of zerumbone for humans.

Altogether, this study demonstrates the antimicrobial activity of zerumbone against *S. mutans*, showing that its toxic activity selectively targets bacterium, but not displays any cytotoxic effect against the normal mammalian cells at concentrations tested. Nonetheless, further studies using in vitro and in vivo models are needed to better determine the zerumbone effectiveness and safety as antimicrobial agent in the context of prophylaxis and treatment of cariogenic infections.

## Conclusions

In summary, this study suggests that zerumbone represents a bioactive substance to be explored by phytopharmaceutical industry in the drug formulations, to prevent and treat cariogenic infections, because this biomolecule showed antimicrobial activity against the main etiological agent of tooth decay, *S. mutans.* Although the cytotoxicity safety of zerumbone in mammalian cells (at concentrations tested) and its antibacterial action at both log and stationary phases support its potential use as antibacterial agent against *S. mutans* infection, additional studies are necessary to better characterize the zerumbone mechanism of action, efficacy and safety in the scenario of cariogenic disease.

## Additional files

Additional file 1: **Figure S1.** Agar BHI showing presence or absence of *S. mutans* CFU representing the MIC and MIB of zerumbone against *S. mutans.* Results are representative of three independent experiments performed in triplicate. Arrows: CFU. (PPTX 454 kb)

Additional file 2: **Figure S2.** Bacterial growth curve of *S. mutans* cells grown in BHI broth for 48 h at 37 in atmosphere of 5% CO_2_ at 37 °C. (PPTX 43 kb)

## Abbreviations

BHI: Brain heart infusion
CFU: Colony forming units
DMSO: Dimethyl sulfoxide
MBC: Mininum bactericidal concentration
MIC: Minimum inhibitory concentration
OD: optical density
RPM: Rotation per minute
