# Supplementary figures and images for: Zerumbone from Zingiber zerumbet (L.) smith: a potential prophylactic and therapeutic agent against the cariogenic bacterium Streptococcus mutans
Source: BMC Complement Altern Med. 2018 Nov 13;18:301. doi: 10.1186/s12906-018-2360-0 (PMC6234655; doi:10.1186/s12906-018-2360-0)

## Slide 1
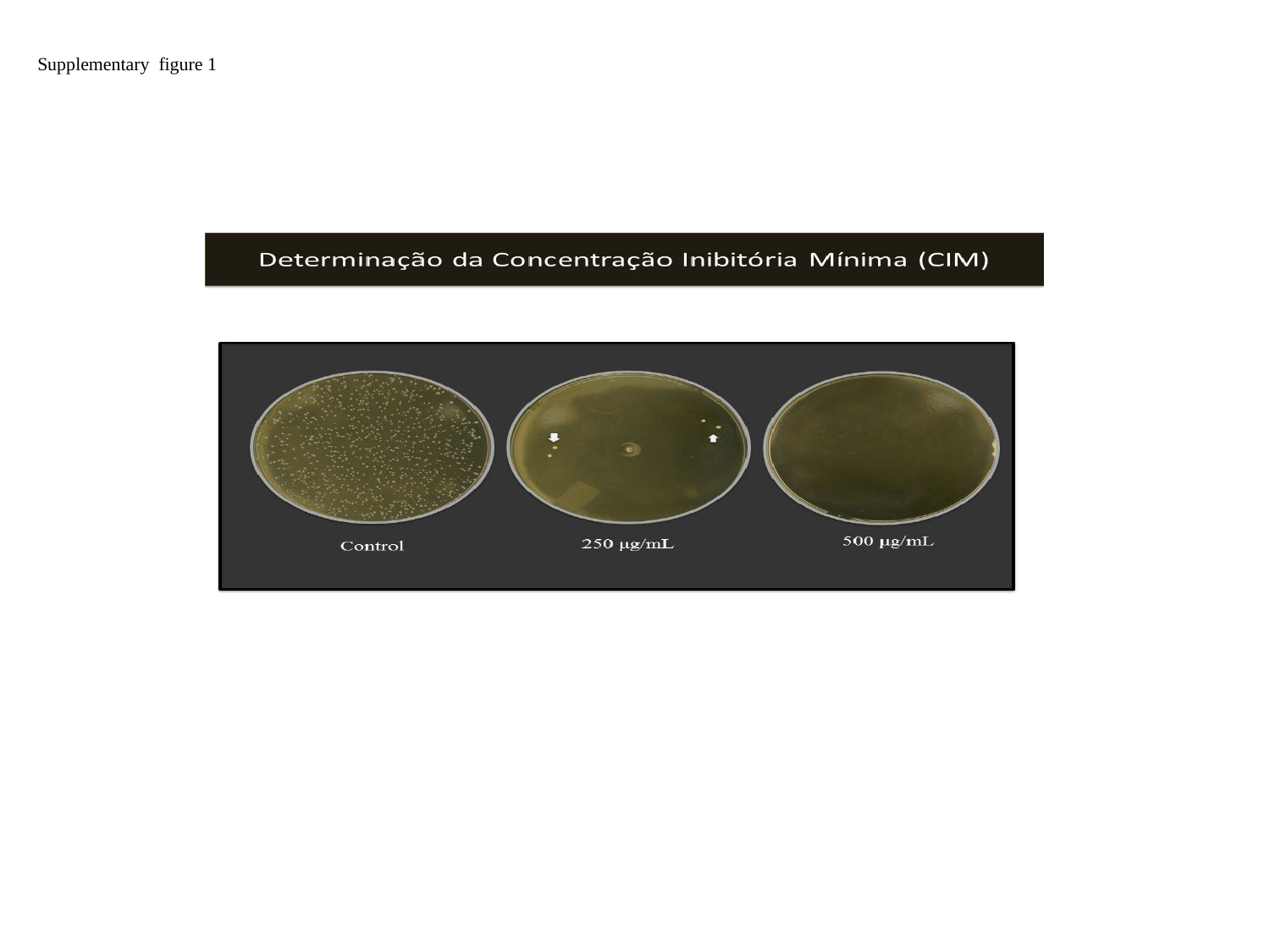

Supplementary figure 1

Supplement: Supplementary file 1 — Figure S1. Agar BHI showing presence or absence of S. mutans CFU representing the MIC and MIB of zerumbone against S. mutans. Results are representative of three independent experiments performed in triplicate. Arrows: CFU. (PPTX 454 kb) [file 12906_2018_2360_MOESM1_ESM.pptx]

## Slide 1
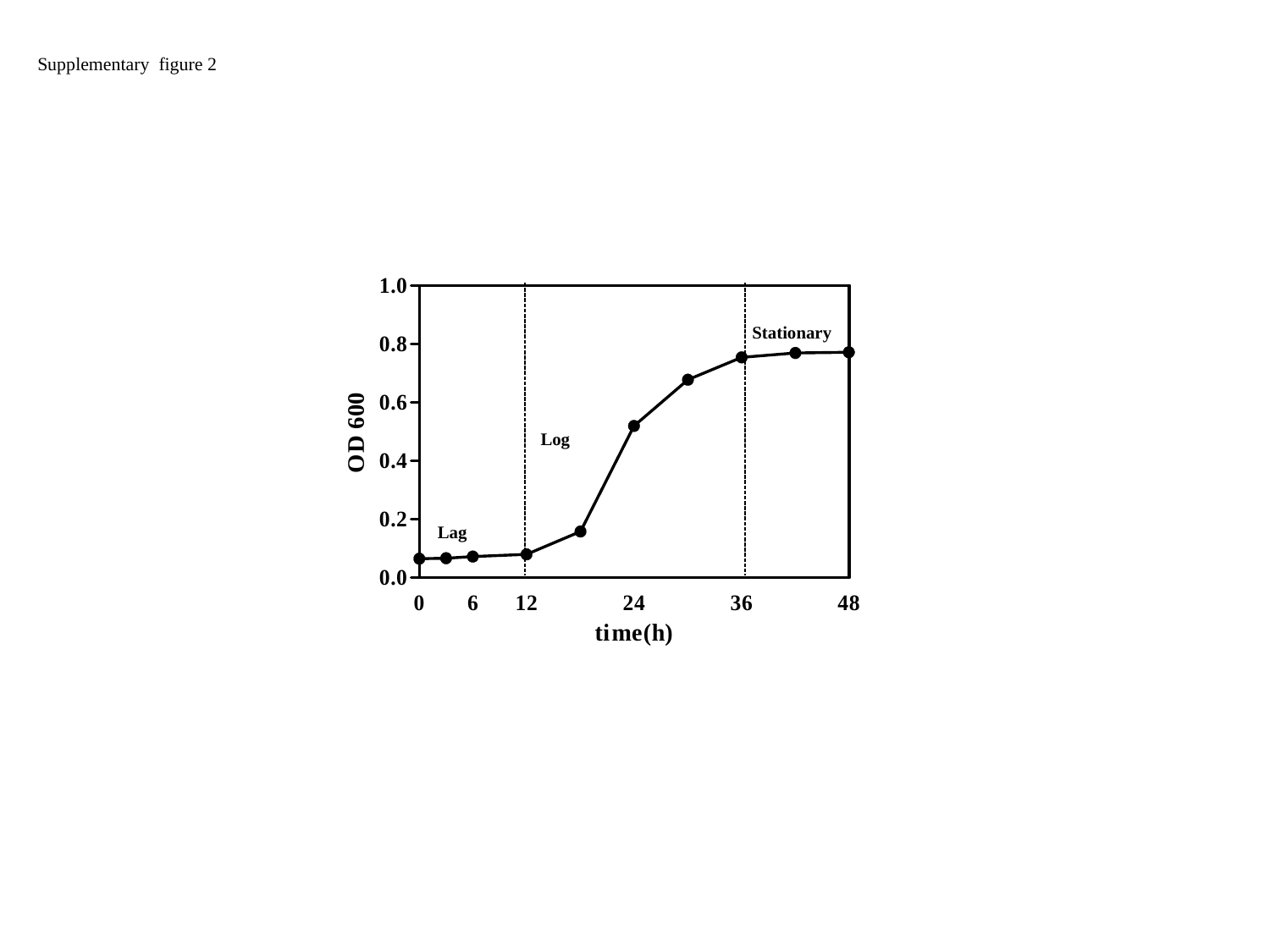

Supplementary figure 2
Stationary
Log
Lag

Supplement: Supplementary file 2 — Figure S2. Bacterial growth curve of S. mutans cells grown in BHI broth for 48 h at 37 in atmosphere of 5% CO2 at 37 °C. (PPTX 43 kb) [file 12906_2018_2360_MOESM2_ESM.pptx]
